# Detection of dengue virus serotype 2 in local *Aedes aegypti* populations, Madeira Island, Portugal, 2025

**DOI:** 10.1186/s13071-026-07251-1

**Published:** 2026-01-27

**Authors:** Líbia Zé-Zé, Vítor Borges, Bruna Raquel Gouveia, Victoria Mary Cox, Manuel Silva, João Dourado Santos, José Alves, Wes Hinsley, Inês Campos Freitas, Daniel Sobral, Rita Fernandes, Fátima Amaro, João Paulo Gomes, Hugo Costa Osório, Nuno Rodrigues Faria, Maria João Alves

**Affiliations:** 1 Department of Infectious Diseases, Centre for Vectors and Infectious Diseases Research (CEVDI), National Institute of Health Doutor Ricardo Jorge (INSA), Águas de Moura, Portugal; 2 Associate Laboratory for Animal and Veterinary Science (AL4AnimalS), Lisbon, Portugal; 3 Genomics and Bioinformatics Unit, Department of Infectious Diseases, National Institute of Health Doutor Ricardo Jorge (INSA), Lisbon, Portugal; 4 Regional Directorate of Health, Funchal, Madeira Portugal; 5 Interactive Technologies Institute – LARSyS, Polo Científico e Tecnológico da Madeira, Funchal, Portugal; 6 MRC Centre for Global Infectious Disease Analysis, School of Public Health, Imperial College London, London, UK; 7 Serviço de Saúde da RAM (SESARAM), Hospital Dr. Nélio Mendonça, Funchal, Madeira Portugal; 8 Center for the Study of Animal Science (CECA), Institute for Agricultural and Agro-Alimentary Science and Technology (ICETA), University of Porto, Porto, Portugal; 9 Animal and Veterinary Research Center (CECAV), Faculty of Veterinary Medicine, Lusófona University – Lisbon University Centre, Lisbon, Portugal; 10 Environment and Infectious Diseases Research Group (ISAMB), Environmental Health Institute, Lisbon, Portugal; 11 Departamento de Moléstias Infecciosas E Parasitárias E Instituto de Medicina Tropical da Faculdade de Medicina, Universidade de São Paulo, São Paulo, Brazil

**Keywords:** Dengue virus, *Aedes aegypti*, Madeira Island, Surveillance

## Abstract

**Background:**

Since 2010, dengue virus (DENV) has caused sporadic outbreaks across Europe, namely in Croatia, Spain, France, Italy and the Portuguese island of Madeira. *Aedes aegypti* mosquito is established in the Autonomous Region of Madeira, and along the eastern Black Sea coast of Cyprus. In Madeira Island, an outbreak of DENV serotype 1 occurred between 2012 and 2013, resulting in 1080 confirmed cases. Despite ongoing entomological surveillance, no further local transmission was detected in the following decade.

**Methods:**

In January 2025, following two suspected dengue cases on Madeira Island, increased entomological surveillance efforts were implemented to confirm a local event transmission of DENV. A network of mosquito traps was complemented by targeted surveillance using 17 BG-PRO traps positioned in the vicinity of suspected human cases. Daily collections of adult *A. aegypti*, collected from 10 January to 31 March 2025, were screened by reverse transcription polymerase chain reaction (RT-PCR) for *Aedes*-borne viruses in the reference laboratory. Viral sequencing was performed using target enrichment and bioinformatics with INSaFLU-TELEVIR. The climate-driven suitability for dengue transmission by *A. aegypti* was also investigated. Serological and molecular tests were conducted on samples from suspected human cases.

**Results:**

Out of 80 analysed *A. aegypti* pools (*N* = 393 mosquitoes), 1 pool, with 9 mosquitoes collected near the home of suspected human cases, tested positive for DENV. The dengue whole genome sequence from this sample was determined and classified as DENV-2 lineage 2II_F.1.1.3. The same virus was retrospectively confirmed in one of the clinical cases. Analysis of mosquito abundance and climate data confirmed the occurrence of this local transmission event during a period of low mosquito abundance and low climatic suitability.

**Conclusions:**

Here, we report an in-depth analysis of a local dengue transmission event that occurred in Funchal, the capital of Madeira Island, in January 2025, with whole-genome evidence of DENV-2II_F.1.1.3 in field-caught *A. aegypti* mosquitoes. Retrospective analysis confirmed the presence of the same virus in one of the two clinical cases, establishing a direct link between human and mosquito infections, and highlighting the risk of off-season arboviral introductions.

**Graphical Abstract:**

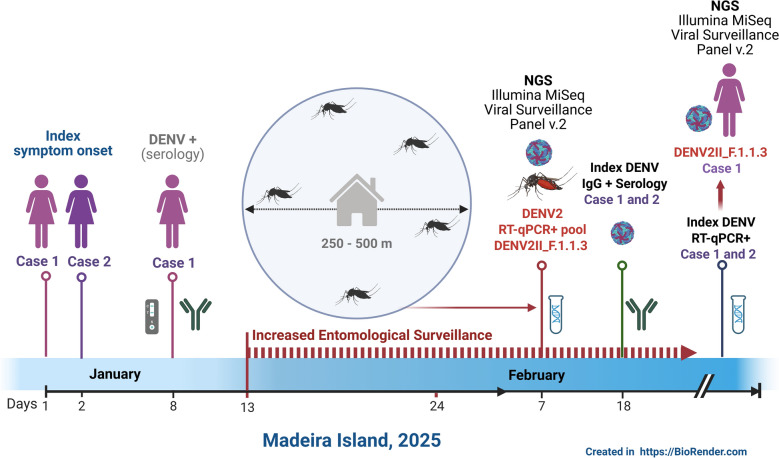

**Supplementary Information:**

The online version contains supplementary material available at 10.1186/s13071-026-07251-1.

## Background

Between 23 September 2012 and 3 March 2013, Madeira Island, Portugal, experienced the largest dengue outbreak ever reported in Europe, with 2168 probable and 1080 confirmed cases [1]. The outbreak was caused by an introduction of dengue virus serotype 1 (DENV-1) (lineage 1V_D.1), closely related to strains circulating in Venezuela [2, 3], and transmitted locally by *Aedes aegypti* mosquitoes, first detected on the island in 2005 [4]. Transmission ceased by March 2013 with no severe cases or fatalities recorded. The establishment of *A. aegypti* populations in Madeira is believed to have resulted from multiple introductions from South America through maritime transportation into the port city Funchal, the island’s capital and major urban centre [5]. Following the 2012–2013 DENV-1 outbreak, *A. aegypti* population densities declined, likely due to community-based vector control interventions [5]. Despite this reduction, *A. aegypti* remained established on the island. Since then, sporadic imported dengue cases have been detected among travellers arriving in Madeira, with no evidence of locally acquired infections. Mosquito surveillance in Madeira has been ongoing since 2005, and since 2010 the Autonomous Region of Madeira has been integrated into of the national vector surveillance network, Rede de Vigilância de Vectores (REVIVE) [6], coordinated by the Centre for Vectors and Infectious Diseases Research (CEVDI), National Institute of Health Doutor Ricardo Jorge (INSA), under the Portuguese Ministry of Health.

In January 2025, following two suspected dengue cases on Madeira Island, entomological surveillance efforts were implemented to confirm local transmission of the DENV. Active public health interventions were implemented to assess the risk of onward transmission of *Aedes*-borne viruses. In the third week of January, entomological investigations confirmed the presence of DENV in mosquitoes captured in Funchal, the capital of Madeira. On 18 February, the Madeira Health Authorities reported the two locally acquired cases [7], following serological confirmation of the two clinical cases and detection of DENV in mosquitoes. Here, we report the in-depth analysis of this dengue local transmission event.

## Methods and results

### Investigation of suspected dengue cases

On 8 January 2025, two related female patients (Case 1, aged 17, and Case 2, aged 52) living in the same household in Funchal attended a private clinic with symptoms compatible with dengue. A lateral flow immunoassay for DENV IgG+IgM+NS1 was performed and yielded a positive result for both patients. Patients were referred to the Public Health and Infectious Disease Department, and the original sample was sent to the local hospital for re-examination. A new lateral flow immunoassay (Rapid dengue fever test, Boson Biotech, China) for DENV IgG + IgM + NS1 confirmed previous results, showing positivity for IgM. A multiplex molecular assay for dengue, chikungunya and Zika viruses (VIASURE® Zika, Dengue & Chikungunya Virus Multiplex PCR Kit, Certest, Spain) was performed on the same samples from both patients, with negative results for all *Aedes*-borne viruses. Sera samples were then sent to the national reference laboratory (CEVDI/INSA) for serological diagnostic confirmation. DENV specific IgM and IgG antibodies [indirect immunofluorescence test (IIFT) Mosaic Dengue virus 1–4 types, Euroimmun] were detected in the Case 1 sample (IgM = 32, cut-off value 16; IgG = 256, IgG cut-off value 32), and both cases were confirmed by IgG seroconversion in samples collected 30 days later (Case 1: IgG = 2028 for DENV-2 and 1024 for DENV-2 and DENV-3; Case 2: IgG = 1024 for DENV-2 and 512 for DENV-1, 2 and 3; Table 1). Neither patient had travelled outside the island of Madeira. Retrospective molecular testing (RealStar® Dengue RT-PCR Kit 3.0, Altona Diagnostics GmbH, Germany), detected low viral loads of DENV RNA in both sera (Case 1: Ct = 36.0; Case 2: Ct = 38.4; Table 1).

**Table 1:** Serologic and molecular diagnostic results

Sera sample	Case 1		Case 2		Assay
	Female, 17 years		Female, 52 years		
Day after symptom onset	7	42	6	41	
*Funchal’s hospital, Madeira Island*					
Lateral flow immunoassay	+IgM	NT	+IgM	NT	Rapid dengue fever test (Boson Biotech, China)
	+IgG		+IgG		
	+NS1		+NS1		
Real-time RT-PCR	—	NT	—	NT	VIASURE® Zika, Dengue & Chikungunya Virus Multiplex PCR Kit (CerTest, Spain)
*Reference laboratory (CEVDI/INSA)*					
Real-time RT-PCR	+Ct = 36.0	NT	+Ct = 38.4	NT	RealStar® Dengue RT-PCR Kit 3.0 (Altona Diagnostics GmbH, Germany)
IIFT IgM^a^	+32	—	—	—	IIFT Mosaic Dengue virus 1–4 types (Euroimmun, Germany)
IIFT IgG^b^	+256	+2048 (DENV2)	—	+1024 (DENV2)	
		+1024 (DENV2 & 3)		+512 (DENV1-3)	

Positive is indicated by “+” and negative by “−”. Ct, Cycle threshold; IIFT, indirect immunofluorescence test; NT, not tested

^a^IgM cut-off value 16

^b^IgG cut-off value 32

### *Aedes aegypti* surveillance and molecular detection

Between 10 January and 31 March 2025, *A. aegypti* were collected within a 250–500 m radius of the cases’ residence using 17 BG-PRO Sentinel traps (Biogents, Germany) additionally deployed (alongside the routinely deployed network with 32 BG traps and 177 ovitraps). Daily collections were performed by trained environmental health technicians from the Autonomous Region of Madeira under the regional surveillance network, with prior consent from property owners. Adult *A. aegypti* mosquitoes were transferred alive into tubes containing RNAlater™ Stabilisation Solution (Invitrogen, Thermo Fischer Scientific, Lithuania) and transported at room temperature to the reference laboratory of entomology in CEVDI/INSA. Species identification was performed using standard morphological keys [8, 9], followed by molecular screening. Ovitraps were inspected weekly to measure both the number of positive ovitraps and the average number of eggs laid by *A. aegypti* females. To increase the number of mosquitoes screened for DENV, eggs were reared in insectary conditions to adulthood. Mosquitoes were grouped into 80 pools based on collection site and homogenised using a refrigerated mortar and pestle with liquid nitrogen, and nucleic acids were extracted using the NUCLISENS® easyMAG platform (Biomérieux, France). Molecular screening for *Aedes*-borne viruses (dengue, Zika and chikungunya) was performed using RealStar® RT-PCR kits (Altona Diagnostics GmbH, Germany) following manufacture’s protocols. DENV typing was conducted using Sanger sequencing of partial C-prM (511 bp) [10] and NS5 (220 bp) amplicons [11].

A total of 393 *A. aegypti* were screened for DENV RNA in 80 pools. Of the pools, 77 contained 234 adult mosquitoes, and 3 of the pools contained 159 mosquitoes that were reared from eggs to adulthood. Females represented 66.9% (*n* = 263) of all analysed mosquitoes (72.2%, *n* = 169/234 of adult collections; 59.1%, *n* = 94/159 of mosquitoes reared from eggs). On 7 February 2025, CEVDI/INSA reported the detection of DENV-2 RNA (Ct = 27.3, RealStar® Dengue RT-PCR Kit 3.0, Altona Diagnostics GmbH, Germany) in a mosquito pool collected in Funchal between 13 and 24 January, near the residence of the suspected human cases. The pool contained nine adult *A. aegypti* (six females, three males). No DENV RNA was detected in pools reared from eggs or in subsequent adult collections from additional ovitraps near the residence during the following 6 weeks. The minimal infection rate (MIR) for January to March 2025 was 4.3% and increased to 15.6% when considering January samples alone.

### Genomic characterisation of DENV in Madeira

Sequencing was performed on an Illumina MiSeq using the Viral Surveillance Panel v.2 (Illumina, EUA), a hybrid-capture target enrichment protocol optimised for the detection and genome sequencing of around 200 viruses, including arboviruses. Reads were quality controlled, screened for viral read and assembled using the INSaFLU-TELEVIR platform v.2.2.0 with default settings [12, 13]. Consensus genome sequences were assembled by reference-guided mapping using a closest match identified by BLASTn [14], and classified with Nextclade v3.13 under updated DENV nomenclature [15, 16]. Phylogenetic analysis was conducted using the Nextstrain workflow [17].

Sequencing of the positive mosquito pool (PTAedesDENV2/P3666/INSA2025) yielded 512,268 reads, of which 23% mapped to DENV-2. A complete consensus genome (10,646 nucleotides; GenBank Accession number: PV748001) was assembled by reference-guided mapping using a closest match (GenBank Accession number PQ357520.1), and classified as lineage DENV 2II_F.1.1.3. For the human samples, 1,448,685 (Case 1) and 885,116 (Case 2) reads were generated in total. Mapping against the DENV-2 genome reconstructed from mosquito pool enabled the identification of 50 closely matching reads in the Case 1 sample (coded as PTHuDENV2/2969/INSA2025).

**Fig. 1:**
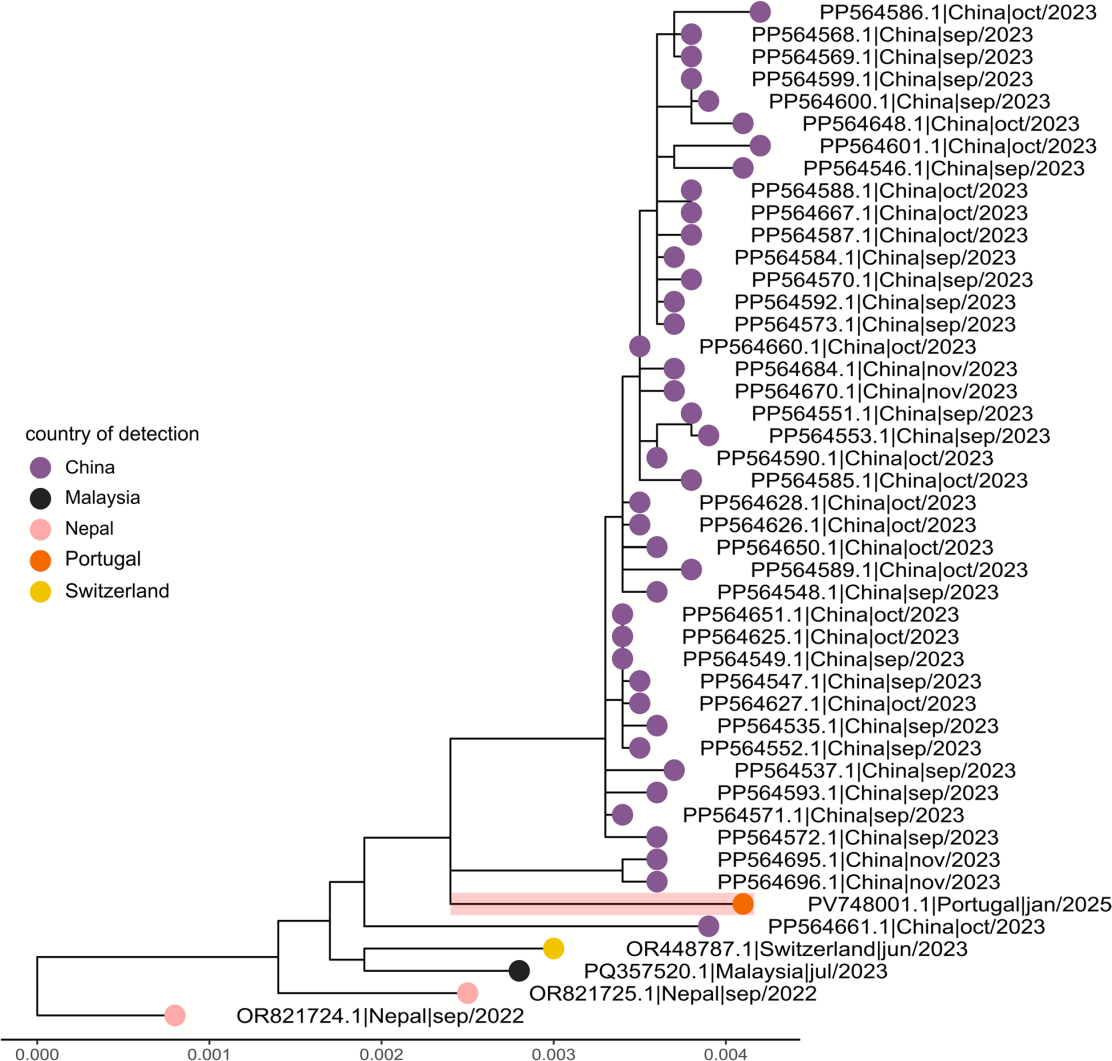
Maximum-likelihood phylogeny of DENV-2 from Madeira (orange) within the 2II_F.1.1.3 lineage (*n* = 46). An extended tree of 1616 genomes is available for interactive exploration on Nextstrain (https://auspice.us/) using the JSON file and metadata provided as a Supplementary Dataset. Branch lengths indicate genetic distance (substitutions per site)

Phylogenetic analysis using the DENV-2 Nextstrain workflow [17] including 1615 complete or near-complete genomes from lineage 2II_F, including 45 from sub-lineage 2II_F.1.1.3 [National Center for Biotechnology Information (NCBI) Virus database, TAXID 11060; accessed 24 April 2025]. The Madeira genome clustered closely with contemporary Asian strains, particularly from China (Fig. 1, Additional file 1: Text S1 and Additional file 2: Dataset S1).

Importantly, the partial sequences from Case 1 were also consistent with classification as DENV-2 lineage 2II_F.1.1.3 (Additional file 3: Fig. S1). No DENV-2 reads were identified in the Case 2 sample.

### Climatic suitability and entomological context

*Aedes aegypti* eggs counts between January 2024 and July 2025, from routine surveillance ovitraps, were used to estimate mosquito abundance. Climatic suitability for arboviral transmission in Madeira in 2024–2025 was estimated using the temperature- and humidity-driven IndexP implemented in the MVSE package [18] with ERA5 meteorological data [19] (Additional file 4: Text S2). Cross-correlation analysis were performed in R software (version 4.4.2).

**Fig. 2:**
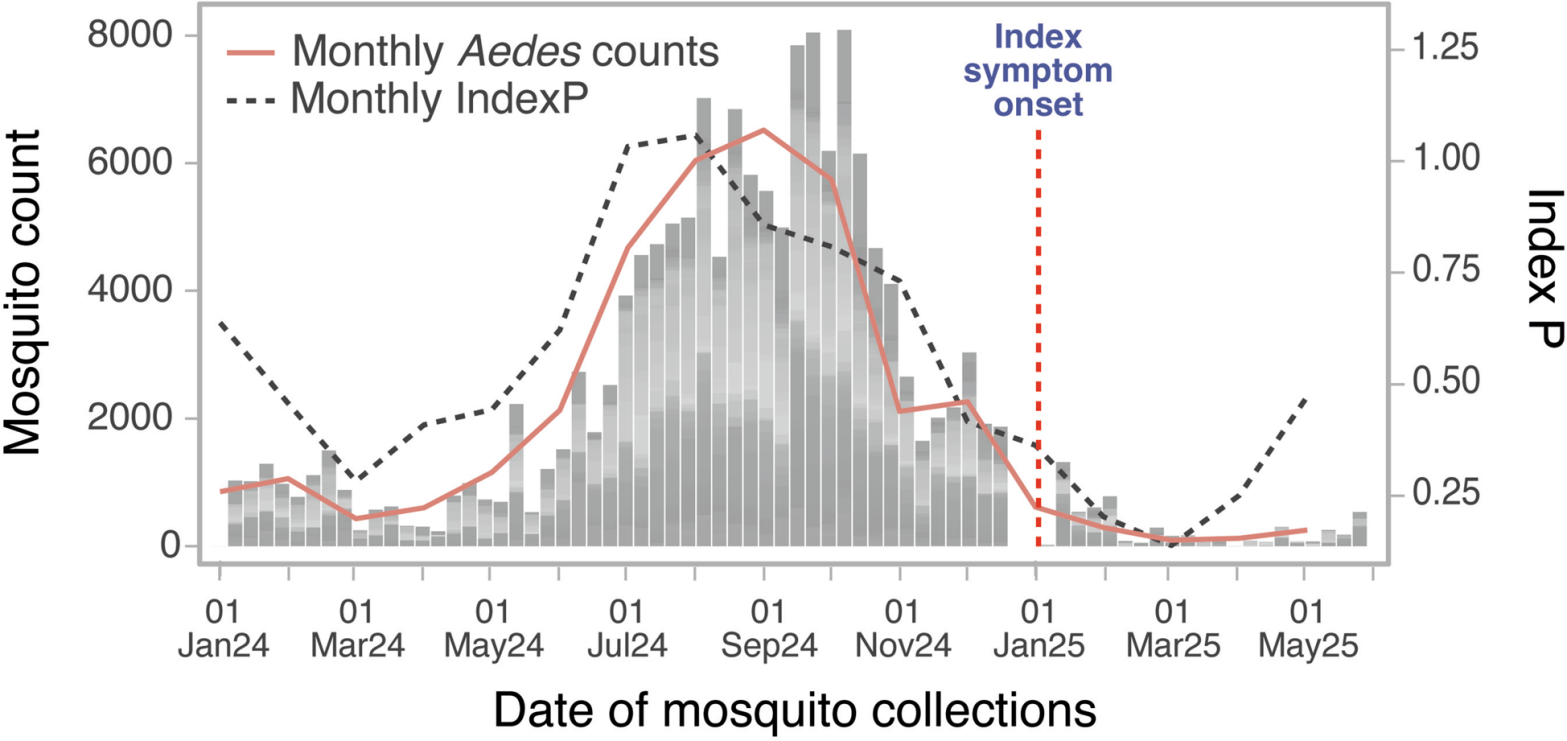
Weekly *A. aegypti* egg counts (grey bars), monthly *A. aegypti* egg counts (salmon line) and monthly climatic transmission suitability (unlagged IndexP, black dashed line) for 2024–2025. Symptom onset of Case 1 is indicated by a red vertical dashed line

A total of 181 ovitraps in 2024 and 177 in 2025, strategically distributed across Madeira including at points of entry at airports and seaports, collected 151,026 *A. aegypti* eggs between January 2024 and July 2025. In 2024, 145,226 *A. aegypti* eggs were collected (mean: 19.5 per ovitrap per week; 69.5 excluding zero-catch weeks). From January to July 2025, 5800 *A. aegypti* eggs were collected (mean: 1.9 per ovitrap per week; 32.4 excluding zero-catch weeks) (Fig. 2). IndexP values above 1 indicate conditions permissive for transmission. Aggregated monthly counts of *A. aegypti* broadly aligned with seasonal peaks in IndexP, which only exceeded 1 between 12 July and 21 August (Fig. 2). Cross-correlation analysis in R (version 4.4.2) revealed that IndexP preceded mosquito abundance by approximately one month (maximum correlation at lag =  −1 month), with regression analysis confirming a strong relationship (lagged model *R*^2^ = 0.82; *P* = 0.0003; unlagged model *R*^2^ = 0.78; *P* = 0.0003). Notably, DENV-2 detection occurred in January 2025, during a period of both low mosquito abundance and low climatic suitability values, suggesting off-season introduction or persistence with little to no onward transmission (Fig. 2).

## Discussion

This study documents the first detection of DENV-2 in Madeira, over a decade after the DENV-1 outbreak in 2012–2013. The virus was identified in a mosquito pool collected near the residence of two human DENV cases with symptom onset in early January 2025. Despite increased entomological surveillance, no additional positive pools or confirmed autochthonous cases were reported up to August 2025, suggesting limited to no onward transmission.

The timeline of events, from symptom onset in early January followed by the detection of DENV-2 in mosquitoes in mid-January aligns with the expected human–mosquito–human transmission cycle. While symptom onset typically occurs 4–10 days after human infection [20], the extrinsic incubation period (EIP) in *A. aegypti* is strongly dependent on temperature [21]. The relatively low average monthly temperature of 18.7 °C recorded in Funchal in January 2025 [22] likely prolonged the EIP. Such cooler temperatures affect mosquito immunity [23], physiology [24] and arbovirus structure and binding [25], collectively reducing transmission potential [26]. It is also possible that *A. aegypti* mosquitoes did not survive long enough to become infectious. Taken together, human and entomological findings suggest that the virus was introduced in late December 2024, consistent with reports of visitors from a dengue-endemic areas staying in the affected residential area during that period.

The detection outside the usual transmission season highlights the possibility of off-season introductions, particularly in areas where *A. aegypti* populations persist year-round, even at low densities. While onward transmission was halted by suboptimal climatic conditions, the event underscores that Madeira remains vulnerable to reintroduction events. With future climate scenarios projecting an overall rise of up to 3.7 °C for Madeira [27], such introductions during winter could lead to onward transmission throughout the year.

Genomic analysis revealed that the virus detected both in mosquitoes and one of the two human cases belonged to DENV-2 lineage 2II_F.1.1.3, clustering with contemporary Asian strains, particularly from China. It is possible that the clustering with strains from China reflects global imbalances in genomic surveillance capacity, with limited sequencing data available for many dengue endemic countries. The 2II_F.1.1.3 lineage is distinct from the Venezuelan DENV-1 lineage associated with the 2012–2013 outbreak, indicating the introduction of a new serotype in Madeira. These findings highlight the island’s vulnerability to future dengue outbreaks sustained by the local *A. aegypti* population and facilitated by international travel and trade. Since 2022, four DENV cases imported from Brazil and Burkina Faso were confirmed on the island [28]. Given Madeira’s role as a tourist hub and port, similar events are likely in the future and will require sustained regional surveillance to detect introductions early and to trigger rapid response interventions. Moreover, the high proportion of susceptible populations to DENV following the 2012–2013 outbreak [29] mean that future introductions may result in larger outbreaks.

Our investigation has several limitations. First, the only human samples available were sera collected several days after symptoms onset. Although these samples were confirmed retrospectively as viraemic, the low viral load of DENV RNA restricted the possibility of retrieving a complete DENV-2 genome. Second, although entomological surveillance was rapidly intensified, the low mosquito abundance during the detection period increased the likelihood that additional positive pools were missed. Nevertheless, while only a single complete viral genome was obtained from mosquitoes, we were able to obtain partial DENV genome reads from Case 1. This was sufficient to confirm that the infection was caused by the same viral lineage (DENV-2 lineage 2II_F.1.1.3) and established a direct link between the human and mosquito infections. The serological results of both cases also indicated that DENV-2 was the most probable cause of the infection, with higher positivity titres. Serological evidence suggests that Case 1 corresponds to a primary DENV infection, while Case 2 is consistent with a secondary infection, supported by the absence of IgM detection.

## Conclusions

The detection of DENV-2 in the *A. aegypti* population from Madeira coincides with a global surge in dengue activity, with over 14.4 million cases reported to the World Health Organization (WHO) in 2024 alone [30]. Climate change increased human mobility, and urbanisation are expanding the geographic range and density of *A. aegypti*, raising the chances of arboviral and mosquito invasion into continental Europe. Our findings highlight the importance of proactive collaborative policies that combine routine entomological monitoring, early warning climate-based systems, genomic characterisation and adaptive vector-control measures. Given that many people infected with *Aedes*-borne viruses remain asymptomatic, all travellers arriving from endemic countries should be encouraged to take preventive measures against mosquito bites for at least 1 week after arriving on Madeira Island. The use of effective insect repellents containing active ingredients such as *N*,*N*-diethyl-*meta*-toluamide (DEET) or picaridin on exposed skin, as well as wearing loose, long-sleeved clothing and long trousers, may play a key role in preventing the introduction of arboviruses to Europe from endemic regions. In continental Europe, these measures may be required primarily on a seasonal basis, particularly in warmer months. However, in Madeira Island, our data indicate that local transmission events can occur all year round. Improving health literacy to enhance compliance with preventive behaviours among all citizens will be essential to mitigate the introduction of arboviruses into previously unaffected areas.

## Supplementary Information

**Additional file 1: Text S1.** Supplementary Dataset S1 files description

**Additional file 2: Dataset S1.** Zipped JSON and metadata TSV files for tree interactive exploration

**Additional file 3: Fig. S1.** Integrative Genomics Viewer (IGV) visualisation of DENV-2 read mapping from the mosquito pool (PTAedesDENV2/P3666/INSA2025) and human Case 1 (PTHuDENV2/2969/INSA2025)

**Additional file 4: Text S2.** Climatic suitability for arboviral transmission estimation

## Data Availability

Reads generated in this study were deposited in the European Nucleotide Archive (ENA) (BioProject accession no. PRJEB87871) under the accession numbers: ERR15107401 (raw reads from mosquito pool; PTAedesDENV2/P3666/INSA2025) and ERR15532383 (DENV2 quality control (QC)-passed reads identified in sample from human Case 1; PTHuDENV2/2969/INSA2025). The DENV2 genome consensus sequence assembled from mosquito pool was submitted to GenBank (accession number: PV748001).

## Abbreviations

DEET: *N*,*N*-diethyl-*meta*-toluamide
DENV: Dengue virus
ECMWF: European Centre for Medium-Range Weather Forecasts
EIP: Extrinsic incubation period
EU: European Union
FCT: Portuguese Foundation for Science and Technology
GIS: Geographic information system
IgG: Immunoglobulin G
IgM: Immunoglobulin M
MIR: Minimal infection rate
NCBI: National Center for Biotechnology Information
NS1: Nonstructural protein 1
RT-PCR: Reverse transcription polymerase chain reaction
WHO: World Health Organization
