# Supplementary material for: Detection of dengue virus serotype 2 in local Aedes aegypti populations, Madeira Island, Portugal, 2025
Source: Parasit Vectors. 2026 Jan 27;19:92. doi: 10.1186/s13071-026-07251-1 (PMC12917965; doi:10.1186/s13071-026-07251-1)
Supplement: Supplementary file 1 — Additional file 1: Text S1. Supplementary Dataset S1 files description [file 13071_2026_7251_MOESM1_ESM.docx]

**Text S1:**

**Supplementary Dataset S1 files description**

The Supplementary dataset S1 files includes the results of the phylogenetic and geotemporal analysis conducted using DENV2 Nextstrain [1] workflow (<https://github.com/nextstrain/dengue>) available at INSaFLU-TELEVIR platform v.2.2.0 ([https://insaflu.insa.pt/](https://eur03.safelinks.protection.outlook.com/?url=https%3A%2F%2Finsaflu.insa.pt%2F&data=05%7C02%7Cvitor.borges%40insa.min-saude.pt%7C6f132481464748ed957b08de07d4b142%7C22c84608f01d46c5802463cc962e5f51%7C0%7C0%7C638956807243864081%7CUnknown%7CTWFpbGZsb3d8eyJFbXB0eU1hcGkiOnRydWUsIlYiOiIwLjAuMDAwMCIsIlAiOiJXaW4zMiIsIkFOIjoiTWFpbCIsIldUIjoyfQ%3D%3D%7C0%7C%7C%7C&sdata=TNcFHHxTOZXmiikZ4CAF0PRdqOfF0sCzTWkBR5EWD4w%3D&reserved=0); [https://github.com/INSaFLU/dengue](https://eur03.safelinks.protection.outlook.com/?url=https%3A%2F%2Fgithub.com%2FINSaFLU%2Fdengue&data=05%7C02%7Cvitor.borges%40insa.min-saude.pt%7C6f132481464748ed957b08de07d4b142%7C22c84608f01d46c5802463cc962e5f51%7C0%7C0%7C638956807243899663%7CUnknown%7CTWFpbGZsb3d8eyJFbXB0eU1hcGkiOnRydWUsIlYiOiIwLjAuMDAwMCIsIlAiOiJXaW4zMiIsIkFOIjoiTWFpbCIsIldUIjoyfQ%3D%3D%7C0%7C%7C%7C&sdata=FwDd%2FySoZWqZparwxDHSId0WnxVxjrd90MzUmEU10vk%3D&reserved=0), as of 2025-05-13) [2,3].

To phylogenetically place the DENV-2 sequence detected in Madeira, Portugal, within the current global genetic diversity of DENV-2, the analysis included 1,615 international complete or near-complete genomes from the major lineage 2II_F (including 45 sequences from lineage 2II_F.1.1.3), available in the NCBI Virus database ([https://www.ncbi.nlm.nih.gov/labs/virus/vssi/#/](https://eur03.safelinks.protection.outlook.com/?url=https%3A%2F%2Fwww.ncbi.nlm.nih.gov%2Flabs%2Fvirus%2Fvssi%2F%23%2F&data=05%7C02%7Cvitor.borges%40insa.min-saude.pt%7C6f132481464748ed957b08de07d4b142%7C22c84608f01d46c5802463cc962e5f51%7C0%7C0%7C638956807243923237%7CUnknown%7CTWFpbGZsb3d8eyJFbXB0eU1hcGkiOnRydWUsIlYiOiIwLjAuMDAwMCIsIlAiOiJXaW4zMiIsIkFOIjoiTWFpbCIsIldUIjoyfQ%3D%3D%7C0%7C%7C%7C&sdata=M0fQEu4or0l5OgtjuDBmj0d82KUB6Yq7tBbpxIbpNKs%3D&reserved=0), TAXID 11060; as of 2025-04-24). DENV clade/lineage classification was performed with Nextclade v3.13 ([https://clades.nextstrain.org/](https://eur03.safelinks.protection.outlook.com/?url=https%3A%2F%2Fclades.nextstrain.org%2F&data=05%7C02%7Cvitor.borges%40insa.min-saude.pt%7C6f132481464748ed957b08de07d4b142%7C22c84608f01d46c5802463cc962e5f51%7C0%7C0%7C638956807243945629%7CUnknown%7CTWFpbGZsb3d8eyJFbXB0eU1hcGkiOnRydWUsIlYiOiIwLjAuMDAwMCIsIlAiOiJXaW4zMiIsIkFOIjoiTWFpbCIsIldUIjoyfQ%3D%3D%7C0%7C%7C%7C&sdata=dKamJKc85fZsSlWZ8l84oOF5ODSW%2BeeiuyKwfXeH260%3D&reserved=0); consulted on 2025-04-24) [4,5].

**The tree can be interactively explored on** [**https://auspice.us/**](https://eur03.safelinks.protection.outlook.com/?url=https%3A%2F%2Fauspice.us%2F&data=05%7C02%7Cvitor.borges%40insa.min-saude.pt%7C6f132481464748ed957b08de07d4b142%7C22c84608f01d46c5802463cc962e5f51%7C0%7C0%7C638956807243966871%7CUnknown%7CTWFpbGZsb3d8eyJFbXB0eU1hcGkiOnRydWUsIlYiOiIwLjAuMDAwMCIsIlAiOiJXaW4zMiIsIkFOIjoiTWFpbCIsIldUIjoyfQ%3D%3D%7C0%7C%7C%7C&sdata=NwBqmQuH6FBq1lQBthx%2BQR3EpBkNAMSzPqgXAr3byQ0%3D&reserved=0) **(first drag&drop both JSON files, and then the metadata TSV file).** Geolocation refers to the country of detection, not necessarily the country of infection.

**References**

1. Hadfield J, Megill C, Bell SM, Huddleston J, Potter B, Callender C, et al. Nextstrain: real-time tracking of pathogen evolution. Bioinformatics. 2018;34:4121-3.
2. Borges V, Pinheiro M, Pechirra P, Guiomar R, Gomes JP. INSaFLU: an automated open web-based bioinformatics suite "from-reads" for influenza whole-genome-sequencing-based surveillance. Genome Med. 2018;10:46.
3. Santos JD, Sobral D, Pinheiro M, Isidro J, Bogaardt C, Pinto M, et al. INSaFLU-TELEVIR: an open web-based bioinformatics suite for viral metagenomic detection and routine genomic surveillance. Genome Med. 2024;16:61.
4. Aksamentov I, Roemer C, Hodcroft E, Neher RA. Nextclade: clade assignment, mutation calling and quality control for viral genomes. J. Open Source Softw. 2021;6: 3773.
5. Hill V, Cleemput S, Pereira JS, Gifford RJ, Fonseca V, Tegally H, et al. A new lineage nomenclature to aid genomic surveillance of dengue virus. PLoS Biol. 2024;22:e3002834.
