# Supplementary material for: Detection of dengue virus serotype 2 in local Aedes aegypti populations, Madeira Island, Portugal, 2025
Source: Parasit Vectors. 2026 Jan 27;19:92. doi: 10.1186/s13071-026-07251-1 (PMC12917965; doi:10.1186/s13071-026-07251-1)
Supplement: Supplementary file 3 — Additional file 3: Fig. S1. Integrative Genomics Viewer (IGV) visualisation of DENV-2 read mapping from the mosquito pool (PTAedesDENV2/P3666/INSA2025) and human Case 1 (PTHuDENV2/2969/INSA2025) [file 13071_2026_7251_MOESM3_ESM.docx]

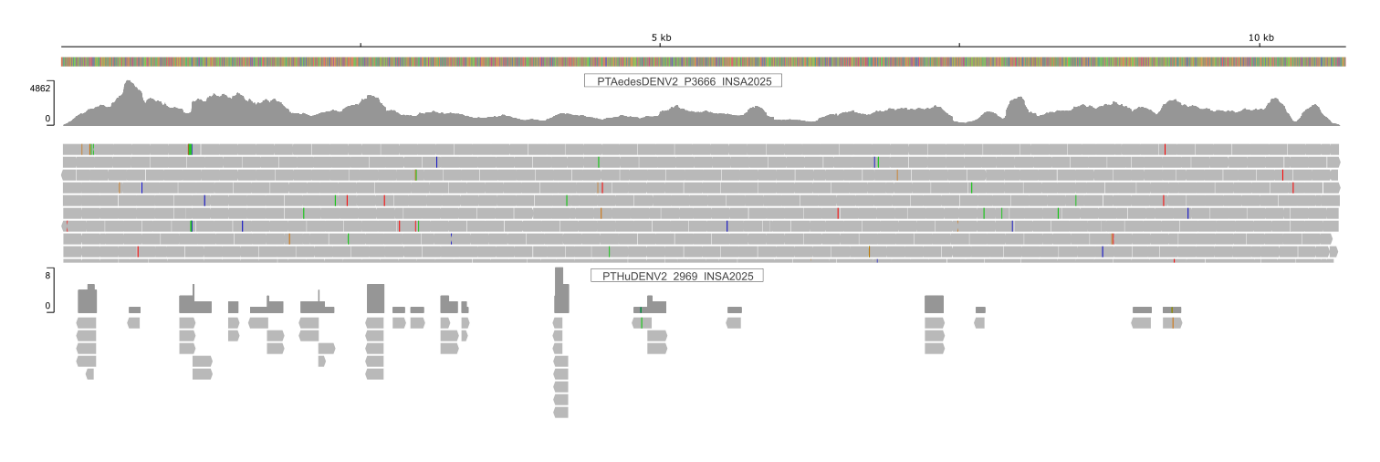


**Figure S1.**Integrative Genomics Viewer (IGV) visualization of DENV2 read mapping from the mosquito pool (PTAedesDENV2/P3666/INSA2025; top) and human case 1 (PTHuDENV2/2969/INSA2025; bottom), using as reference the DENV2 consensus sequence reconstructed from the mosquito sample (accession PV748001). The 50 DENV reads identified in the human sample closely matched the mosquito-derived genome, were well distributed across the genome, and confirmed nucleotide substitutions specific to lineage 2II_F.1.1.3 (e.g., G1599T), as well as mutations segregating the Madeira DENV2 from other genomes within the same lineage (e.g., C1197T, C2064T, A2262G).
