# Supplementary material for: Detection of dengue virus serotype 2 in local Aedes aegypti populations, Madeira Island, Portugal, 2025
Source: Parasit Vectors. 2026 Jan 27;19:92. doi: 10.1186/s13071-026-07251-1 (PMC12917965; doi:10.1186/s13071-026-07251-1)
Supplement: Supplementary file 4 — Additional file 4: Text S2. Climatic suitability for arboviral transmission estimation [file 13071_2026_7251_MOESM4_ESM.docx]

**Text S2:**

**Climatic suitability for arboviral transmission estimation**

Global hourly data for 2m dew point temperature and 2m mean temperature between 1 January 2024 to 31 May 2025 were downloaded from the ERA5-Land reanalysis dataset (ERA5-Land hourly data from 1950 to present) provided by Copernicus Climate Change Service at 0.1 x 0.1 degrees resolution [1]. Relative humidity ($RH$) was calculated using the dew point ($d$) and temperature ($T$) following the August‑Roche‑Magnus formula:

$$RH=100*\frac{exp(d*17.625)/(d+243.04)}{exp(T*17.625)/(T+243.04)} .$$

The points within Madeira Island were sampled, categorised using Portuguese administrative borders specified by a GADM shapefile [2]. The surface area of each point grid square, which varies by latitude, was used to calculate the weighted average of the mean daily temperature and mean daily relative humidity.

IndexP, a measure of *Aedes* transmission potential, was calculated using the temperature and humidity data and the MVSE (Mosquito-borne Viral Suitability Estimator) R package [3]. MVSE estimates IndexP using a mechanistic transmission model implemented in a Bayesian Markov Chain Monte Carlo (MCMC) framework. The following priors were assumed for the model parameters, mosquito life expectancy (days): mean 14 and standard deviation 3, mosquito incubation period (days): mean 7 and standard deviation 2, mosquito biting frequency (bites/female/day): mean 0.25 and standard deviation 0.05, human life expectancy (years): mean 73 and standard deviation 2, human incubation period (days): mean 5 and standard deviation 1, human infectious period (days): mean 5 and standard deviation 1, and human-mosquito transmission probability: mean 0.5, standard deviation 0.01. All parameters were assumed to follow a Gaussian distribution. MCMC was run for 100,000 steps and the posterior distributions were sampled 1,000 times. Daily IndexP estimates were aggregated to calculate monthly averages.

**References**

1. Muñoz Sabater J. ERA5-Land hourly data from 1950 to present. Copernicus Climate Change Service (C3S) Climate Data Store (CDS). 2019.
2. GADM. Global Administrative Areas. GADM database of Global Administrative Areas, version 2.0. (2018). <https://gadm.org/>. Assessed: 20 August 2025.
3. Obolski U, Perez PN, Villabona-Arenas CJ, Thézé J, Faria NR, Lourenço J. MVSE : An R-package that estimates a climate-driven mosquito-borne viral suitability index. Methods Ecol Evol. 2019;10:1357–70.
